# Borderline tuberculoid leprosy misdiagnosed as Cheilitis Granulomatosa Miescher: A case report

**DOI:** 10.1002/ccr3.6240

**Published:** 2022-08-09

**Authors:** Seema Sitaula, Aman Mishra, Sanat Chalise, Aakash Mishra

**Affiliations:** ^1^ Department of Dermatology and Venereology, Maharajgunj Medical Campus Tribhuvan University Institute of Medicine Kathmandu Nepal; ^2^ Maharajgunj Medical Campus Tribhuvan University Institute of Medicine Kathmandu Nepal; ^3^ Department of Pathology Kathmandu Medical College Teaching Hospital Kathmandu Nepal; ^4^ Kathmandu Medical College Teaching Hospital Kathmandu Nepal

**Keywords:** Cheilitis Granulomatosa Miescher, leprosy, lip swelling, macrocheilia, *Mycobacterium leprae*

## Abstract

Macrocheilia, as an initial manifestation of leprosy, is uncommon. We present a case of a 50‐year‐old female, with lower lip swelling, initially diagnosed as Cheilitis Granulomatosa Miescher. Unresponsiveness to local intralesional corticosteroids necessitated further evaluation. Repeat tissue sampling yielded a confirmatory diagnosis of borderline tuberculoid leprosy, which was managed successfully.

## Graphical abstract

Borderline tuberculoid leprosy should be considered as a differential diagnosis of chronic macrocheilia in countries with a high disease burden. A high index of suspicion, microbiological, and histological tests is required for an accurate diagnosis.

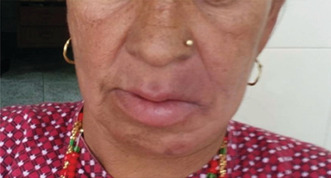

## 1 INTRODUCTION

Isolated Cheilitis Granulomatosa (CG) is called Cheilitis Granulomatosa Miescher (CGM) which is characterized by recurrent firm swelling of one or both lips for more than 8 weeks and an isolated granulomatous macrocheilitis on histology.^1^, ^2^ Leprosy or Hansen's disease, caused by *Mycobacterium leprae* (*M. leprae*) affects the skin and peripheral nerves primarily.^2^, ^3^ Oral‐mucosal involvement is rare in tuberculoid and borderline forms. We report a case of borderline tuberculoid leprosy presenting with chronic lip swelling for 2 years. Chronic macrocheilia as a presenting feature of borderline tuberculoid leprosy has been reported in a handful of reports in the literature, making our case report extremely rare.^2^, ^4^, ^5^

## 2 CASE REPORT

A 50‐year‐old female presented to the outpatient department with persistent swelling of the lower lip of 2 years' duration. She neither reported a history of tuberculosis nor contact with active tuberculosis or leprosy patients.

Routine blood biochemistry and hematological parameters of the patient were within normal limits. Slit skin smear (SSS) examination, Mantoux test, and chest radiograph were unremarkable. Hence, based on the clinical features and investigation, a diagnosis of CGM was made and multiple dosages of intralesional corticosteroid were injected into the lower lip. However, the patient did not respond to the treatment.

**FIGURE 1:**
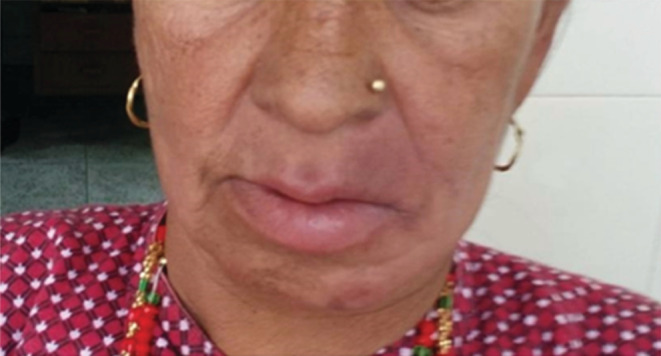
Pretreatment photograph showing lower lip edema and erythema with an infiltrative plaque over the right lower chin and lateral border of the upper lip.

**FIGURE 2:**
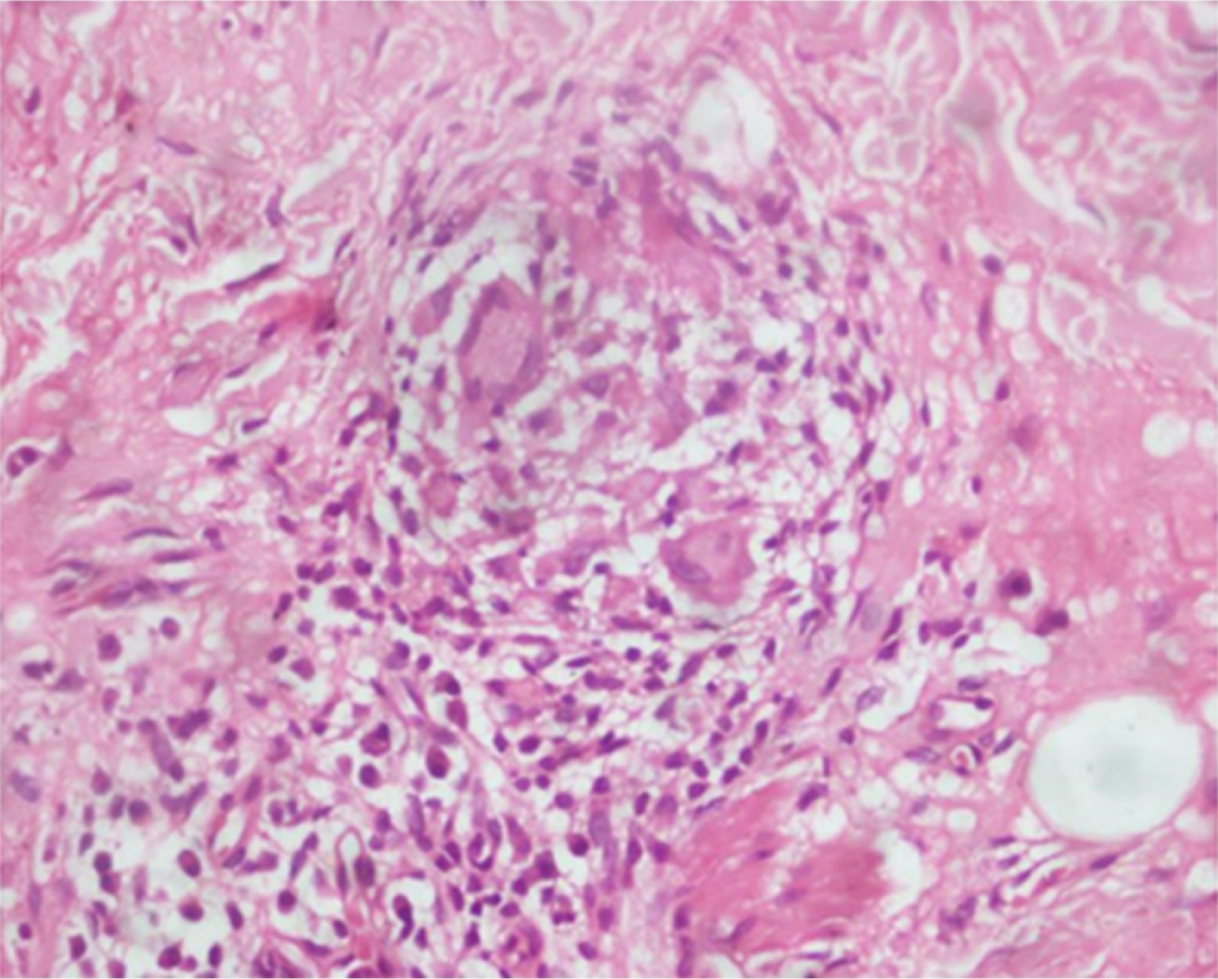
Granuloma in the dermis around periadenexal stucture (HPE 100× magnification).

**FIGURE 3:**
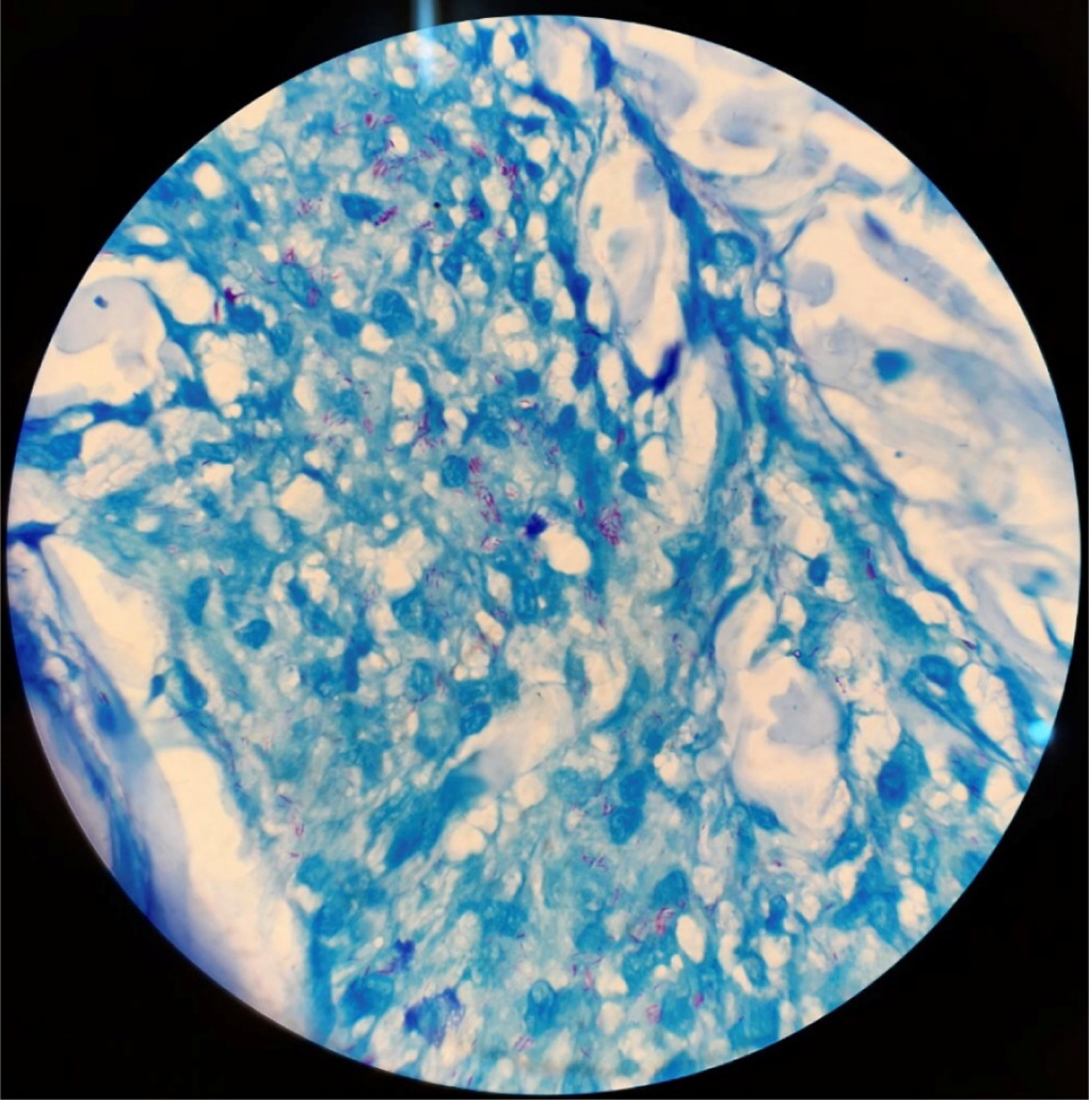
Acid‐fast bacilli in the tissue section using Wade‐Fite stain (HPE oil immersion 1000× magnification).

Four weeks later, she presented with an erythematous, non‐pruritic, non‐scaling plaque above her upper lip and a similar plaque on the right lower chin, each lesion measuring approximately 1 cm × 1.5 cm (Figure 1). Systemic examination did not reveal any abnormalities. An incisional biopsy of the lower lip and the erythematous plaque was done which revealed granuloma formed by epithelioid histiocytes with periadnexal and perineural lymphocytic infiltration on histopathological examination (HPE) (Figure 2). Special stains done for *M. leprae* revealed weak acid‐fast bacilli (Figure 3).

**FIGURE 4:**
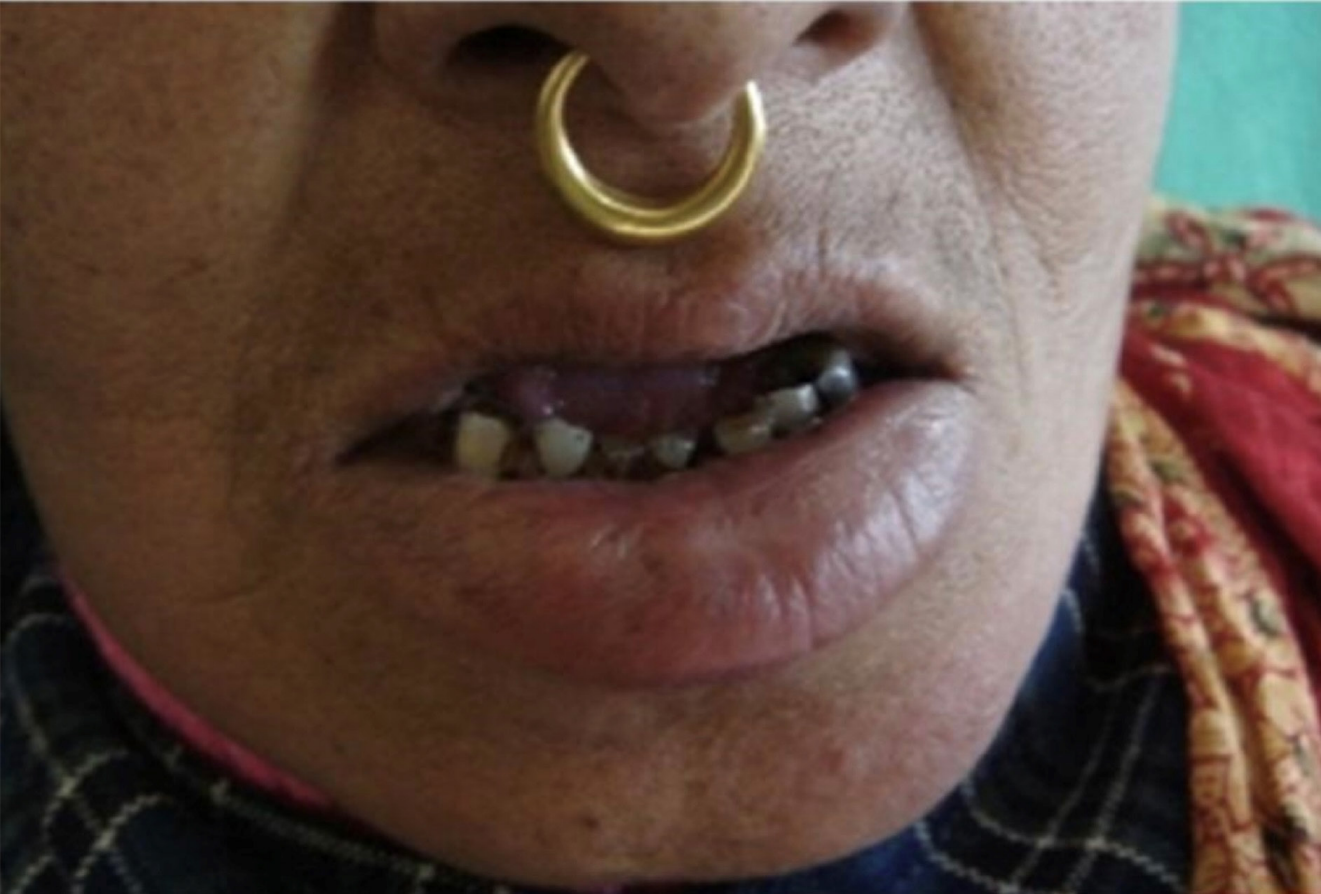
Post‐treatment photograph after 6 months showing resolved lesion.

A final diagnosis of borderline tuberculoid leprosy was made. She was started on the World Health Organization multibacillary multidrug therapy (MB‐MDT) for 12 months and kept on regular follow‐up. The lesions improved with medication (Figure 4).

## 3 DISCUSSION

Leprosy, a chronic infectious granulomatous condition, is caused by *M. leprae*. Leprosy is endemic in Nepal. In 2018, there were 3249 cases reported, and a significant transmission was notable in the pediatric population.^6^ The disease course and spectrum of clinical manifestations are determined by individual cell‐mediated immunity of the host, the most severe form is lepromatous leprosy (LL), where the patient has a compromised cell‐mediated immunity and tuberculoid leprosy (TL), being the mildest one.^3^

Oral‐mucosal involvement has been reported in 20%–60% of patients with LL, where tongue, anterior hard palate, lips, and vulva are the most frequently affected sites.^1^, ^2^ The oro‐mucosal involvement is a common late manifestation in LL; however, oro‐mucosal lesions are rare in the tuberculoid and borderline spectrum.^2^, ^5^, ^7^

Leprosy, presenting as lip swelling, is rare with only a few cases reported in the literature. Handa et al.^2^ reviewed 28 patients with chronic macrocheilia and only three patients had leprosy, describing leprosy as the third most common and eminent cause of macrocheilia after CG and tuberculosis. In our case, the patient presented with swelling of the lip and was diagnosed as a borderline tuberculoid form of leprosy. Based on the clinical presentation, the three major differential diagnoses were CG, tuberculosis, and leprosy. CG is a rare disease, most commonly seen in adults with peak incidence reported between 20 and 40 years of age.^1^, ^2^ CG along with relapsing facial nerve palsy and lingua plicata forms the classical triad of Melkersson–Rosenthal syndrome (MRS).^1^, ^2^ CGM presents with isolated painless persistent swelling of one or both lips as seen in our case.

Leprosy presenting with chronic macrocheilia can be misdiagnosed as MRS. Waxing and waning pattern of lip swelling is seen in both CG and leprosy.^2^ Facial palsy may develop in both MRS as well as leprosy as paralysis of the trigeminal nerve has been noted in leprosy patients. But the characteristic fissured tongue if present is diagnostic of MRS.^1^, ^7^, ^8^ In leprosy, the skin lesion associated with swelling of the lips is warm, tender, scaly and the edge of the lesion extends beyond the lip which are minute distinguishing features from CG.^2^ These features were absent in our case.

Differentiating chronic macrocheilia due to leprosy from CG establishes the significance of performing a skin biopsy with histopathological study.^1^, ^8^ Histologically, both CG and leprosy are characterized by the presence of non‐caseating granuloma with multinucleated Langhans‐type giant cells. CG is further marked by perivascular mononuclear infiltration, lymphedema, and fibrosis. Borderline lepromatous and lepromatous leprosy is characterized by granuloma in the upper dermis and infiltrates composed of foamy cells, lymphocytes, and plasma cells present mostly around the nerves and appendages, distinguishing it from CG.^1^, ^8^

Our patient presented with isolated lip swelling, without tongue and facial nerve involvement. The HPE features were suggestive of CG with SSS negative for *M. leprae*, a diagnosis of CGM was made initially and treated with intralesional corticosteroids. However, there were no signs of clinical improvement and development of new plaques instead which warranted further investigation. A re‐biopsy from the plaque was done, which revealed weakly acid‐fast bacilli on Wade‐Fite stain, suggestive of *M. leprae*. Hence, the diagnosis of borderline tuberculoid leprosy was based on repeated sampling and biopsies on the background of a high index of clinical suspicion.

## 4 CONCLUSION

We presented an unusual case of borderline tuberculoid leprosy presenting as chronic macrocheilia. Lip swelling is an atypical and rare clinical manifestation of tuberculoid and borderline leprosy and should be considered as a differential diagnosis of chronic macrocheilia in countries like Nepal where the disease burden is still significant. A high index of suspicion, microbiological, and histopathological studies is of utmost importance in establishing the correct diagnosis.

## AUTHOR CONTRIBUTIONS

SS conceptualized the study, edited the manuscript, and contributed to the treatment of the patient. AmM conceptualized the study, prepared the original draft. SC contributed to histopathological studies and edited the manuscript. AaM revised the manuscript.

## FUNDING INFORMATION

None.

## CONFLICT OF INTEREST

Nothing to state.

## ETHICAL APPROVAL

Not required.

## CONSENT

Written informed consent was obtained from the patient for the publication of this case report and accompanying images.

## Data Availability

The data that support the findings of this study are available from the corresponding author upon reasonable request.
